# Metallurgical Thermodynamic Design Research on the In Situ Synthesis of Ti-Al-Nb Alloys Using Thermit Self-Propagating Reduction

**DOI:** 10.3390/ma19091689

**Published:** 2026-04-22

**Authors:** Han Jiang, Tingan Zhang, Zhihe Dou

**Affiliations:** 1 School of Metallurgy, Northeastern University, Shenyang 110819, China; jiangh14@mails.neu.edu.cn; 2 Key Laboratory of Ecological Metallurgy of Multi-Metal Intergrown Ores of Ministry of Education, Shenyang 110819, China

**Keywords:** thermodynamic design, titanium–aluminum alloys, aluminothermic reduction, self-propagating metallurgy

## Abstract

Based on the thermodynamic design of metallurgical reduction, this paper investigates the thermodynamic principles and reaction regulation mechanism of aluminothermic self-propagating reduction for the in situ synthesis of a Ti_45_Al_8_Nb (at%) titanium–aluminum–niobium alloy. The influence of the aluminum distribution coefficient (ADC) on the self-propagating reaction process was verified via high-temperature thermal state experiments. The results show that the thermodynamically predicted trends of phase composition and alloy composition are consistent with the experimental results, with only a ~20% lateral offset in the ADC. When the ADC is set to 0.8, the mass fractions of Ti, Al, Nb, O, and N in the alloy are 51.8%, 29.5%, 17.4%, 1.2%, and 0.0016%, respectively, with a homogeneous microstructure and inclusion size no larger than 8 µm. The alloy presents a typical coarse-grained structure, where 83.1% of the total grain boundary length is low-angle grain boundaries, and the <111> orientation is dominant. A low-energy coherent interface is formed between the Ti-enriched and Nb-enriched regions by TiAl, TiAl_3_ and Al_3_Nb phases, which enhances the structural stability of the alloy.

## 1. Introduction

Next-generation high-temperature structural materials are required to be “stronger and lighter” to withstand extreme service environments [1]. Titanium–aluminum alloys, with low density, high specific strength and excellent high-temperature mechanical properties, are widely used in aerospace, marine engineering and medical devices, and are recognized as one of the most promising candidate materials for aero and automotive engines [2,3]. However, their limited room-temperature deformability and insufficient oxidation resistance above 800 °C have long restricted their further development and application [4]. The addition of niobium (Nb) can effectively improve the room-temperature plasticity, creep strength and high-temperature corrosion resistance of titanium–aluminum alloys, significantly expanding their application value and scope [5,6].

Ingot metallurgy (IM) and powder metallurgy (PM) are still the main preparation methods for titanium–aluminum-based alloys [7,8]. However, due to the high melting point of Nb, segregation and phase inhomogeneity are prone to occur during the preparation of titanium–aluminum–niobium alloys via ingot metallurgy [9,10]. Powder metallurgy has significant advantages in the preparation of ultrafine-grained titanium–aluminum-based materials compared with ingot metallurgy [11,12]. Chen et al. [13] pioneered the preparation of Ti_45_Al_10_Nb0.1Y and Ti_48_Al_18_Nb0.1Y alloys via powder metallurgy composite melting, which increased the service temperature of titanium–aluminum alloys by 100 °C. Li et al. [14] prepared high-Nb-TiAl alloys via alloying technology combined with magnetic levitation stirring–consumable electrode arc melting, and further optimized the properties of the master alloy by adding C, W, B and other elements. Li et al. [15] fabricated Al_2_O_3_-reinforced high-Nb-TiAl laminated composites via direct current magnetron sputtering combined with foil-to-foil metallurgy. The composite process of spark plasma sintering (SPS) and mechanical alloying (MA) using metal powders has also attracted extensive attention from scholars [16,17,18,19,20,21]. Titanium–aluminum–niobium alloys prepared using this method contain columnar strengthening phases and exhibit excellent fracture toughness. Lahiri A et al. [22] prepared an Al_3_Ti alloy via electrodeposition using 1-ethyl-3-methylimidazolium chloride (EmimCl) and anhydrous AlCl_3_ as the electrolyte with a double titanium electrode. The Fray–Farthing–Chen (FFC) process uses metal oxides as cathode precursors and graphite rods (or inert electrodes) as anodes to directly convert metal oxides into metals or alloys. Although this process has significant economic advantages, it is still difficult to achieve large-scale industrial production [23,24,25].

Therefore, developing low-cost preparation technology for titanium–aluminum-based alloys is still a key challenge and research focus in the titanium industry. The core breakthrough lies in replacing the long process of sponge titanium production and reducing the energy consumption caused by repeated remelting in the subsequent alloy preparation process. To address this issue, Song et al. [26] at Northeastern University proposed an aluminothermic self-propagating synthesis process for the in situ preparation of titanium alloys and successfully prepared TiAl alloy ingots. This process has the advantages of high efficiency and low cost, but there are still challenges in the precise control of alloy composition and impurity distribution.

In this paper, the metallurgical thermodynamic design of the reaction system was carried out to investigate the influence of aluminum-to-titanium ratio on the reaction process and product quality. By clarifying the thermodynamic mechanism governing the actual reaction results, a controllable and efficient breakthrough was achieved in the preparation of titanium–aluminum–niobium alloys via self-propagating reduction–displacement synthesis.

## 2. Experimental Methods

### 2.1. Experimental Materials and Procedures

The raw materials used in this study, along with their specific purities and manufacturers, are detailed in Table 1.

**Figure 1:**
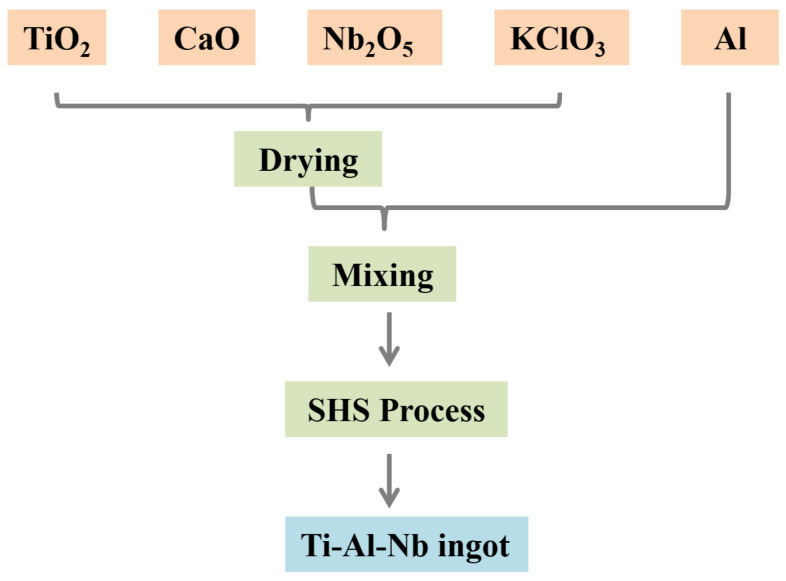
Schematic diagram of the experimental procedure for the preparation of the Ti-Al-Nb alloy.

A schematic diagram of the process is shown in Figure 1. The raw materials (Table 1) were prepared by drying all components except aluminum powder in a blast oven at 100–120 °C for over 4 h to eliminate adsorbed water. The dried materials were then weighed according to the proportions in Table 2 and homogenized in a mixing tank with rubber balls for 20 min to ensure a uniform chemical and physical distribution.

The resulting mixture was then placed into a high-purity graphite crucible reactor and manually stacked into a conical shape. The self-propagating reaction was initiated by igniting a small quantity of magnesium powder placed at the apex of the mixture. Upon ignition, the reaction wave propagated rapidly and spontaneously through the entire charge. After the reaction reached completion and the system was allowed to cool naturally to room temperature, the reactor was inverted to separate the metallic alloy ingot from the slag phase [23].

For compositional analysis, representative portions of the alloy were crushed and pulverized using a hammer, and the resulting powder was sieved through a 100-mesh screen to ensure a fine and uniform sample for subsequent testing. For microstructural characterization, the alloy samples were cut into 10 mm cubes using a wire-electrode cutting machine, followed by standard grinding and polishing procedures.

### 2.2. Analysis and Characterization

Phase characterization of the samples was performed using an X-ray diffractometer (XRD, D8 ADVANCE, Bruker, Bremen, Germany). The chemical compositions were determined via an inductively coupled plasma optical emission spectrometer (ICP-OES, Optima 8300, PerkinElmer, Shelton, CN, USA) and an oxygen–nitrogen analyzer (G8 GALILEO, Bruker, Germany). The microstructural evolution and elemental distribution were characterized using scanning electron microscopy (SEM, Zeiss, Oberkochen, Germany) equipped with energy dispersive spectroscopy (EDS). Electron backscatter diffraction (EBSD, Symmetry S3, Oxford Instruments, Abingdon, UK) was performed to analyze the grain boundary characteristics and texture evolution. Further detailed microstructural analysis was conducted using transmission electron microscopy (TEM, Zeiss, Oberkochen, Germany).

## 3. Results and Discussion

### 3.1. Thermodynamic Design

To systematically investigate the influence of the reducing agent and alloying element addition on the self-propagating process, the concept of the aluminum distribution coefficient (ADC) is introduced. The ADC is defined as the mass ratio of the actual aluminum added to the reaction mixture (*m_Al_*_(*actual*)_) versus the theoretical aluminum mass required (*m_Al_*_(*theoretical*)_) to achieve the designed target alloy composition and is calculated using Equation (1):

(1) $$\mathrm{ADC}\;=\;\frac{m_{\mathrm{Al}(\mathrm{actual})}}{m_{\mathrm{Al}(\mathrm{theoretical})}}$$

Based on Equation (1), an ADC of 1.0 represents the exact stoichiometric proportion required for both the complete reduction of the metal oxides (TiO_2_ and Nb_2_O_5_) and the formation of the target Ti_45_Al_8_Nb alloy matrix. However, considering the deviation between actual and theoretical values, we need to correct the ADC to obtain alloys with qualified compositions in practical applications.

According to the composition requirements of the Ti_45_Al_8_Nb (at%) target alloy, the reaction system shown in Equation (2) was designed:

TiO_2_ + 0.085Nb_2_O_5_ + 2.574Al = Ti + 0.957Al + 0.17Nb + 0.809Al_2_O_3_ (2)

Calculations based on relevant thermodynamic data [27] indicate that the adiabatic temperature (*T_ad_*) for Equation (2) is 2103 K. The detailed thermodynamic derivation process for this value is provided in the Supplementary Material. Here, *T_ad_* refers to the maximum temperature the system can reach under the condition of no heat exchange with the environment, where the temperature rise depends solely on the heat released by the self-propagating reaction. According to the empirical criterion for self-propagating high-temperature synthesis (SHS), the combustion wave can only be sustained spontaneously without external energy input when the *T_ad_* of the system is strictly greater than 1800 K [28,29]. Since the calculated *T_ad_* (2103 K) well exceeds this critical threshold, it thermodynamically guarantees that the designed reaction system can successfully proceed via a self-sustaining self-propagating chemical process [30,31,32].

In this study, all thermodynamic calculations were conducted under standard conditions: pure substances, 298.15 K, and 101,325 Pa.

**Figure 2:**
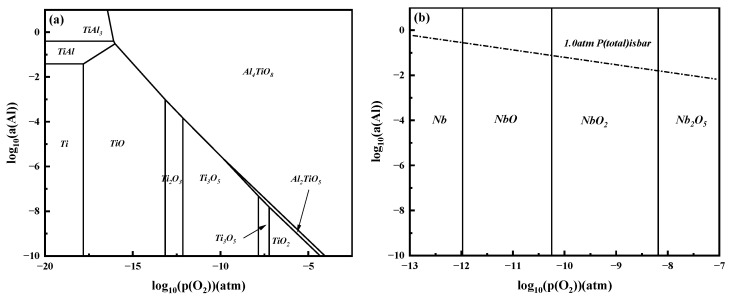
Map of dominant zones of Ti-Al-O (**a**) and Nb-Al-O (**b**) at the designed reaction temperature of 2100 K.

As illustrated in Figure 2, the thermodynamic advantage zone diagrams for the Ti-Al-O and Nb-Al-O systems reveal the stepwise reduction pathways for the respective metal oxides. By analyzing the stability regions of various sub-oxides at the specified reaction temperatures, the sequential reduction processes are determined and represented by Equations (3) and (4).

TiO_2_ → Ti_3_O_5_ → Ti_2_O_3_ → TiO → Ti (3)

Nb_2_O_5_ → NbO_2_ → NbO → Nb (4)

Based on the designed thermodynamic reaction system, it can be seen that the reduction reactions in Equations (5)–(13) may occur during the actual reaction process:

TiO_2_ + 1.33Al = Ti + 0.67Al_2_O_3_ (5)

TiO_2_ + 0.67Al = TiO + 0.33Al_2_O_3_ (6)

TiO_2_ + 0.333Al = 0.5Ti_2_O_3_ + 0.167Al_2_O_3_ (7)

TiO_2_ + 0.222Al = 0.333Ti_3_O_5_ + 0.111Al_2_O_3_ (8)

TiO_2_ + 0.167Al = 0.25Ti_4_O_7_ + 0.0833Al_2_O_3_ (9)

TiO + 0.667Al = Ti + 0.333Al_2_O_3_ (10)

Nb_2_O_5_ + 3.33Al = 2Nb + 1.67Al_2_O_3_ (11)

Nb_2_O_5_ + 2Al = 2NbO + Al_2_O_3_ (12)

NbO + 0.667Al = Nb + 0.333Al_2_O_3_ (13)

**Figure 3:**
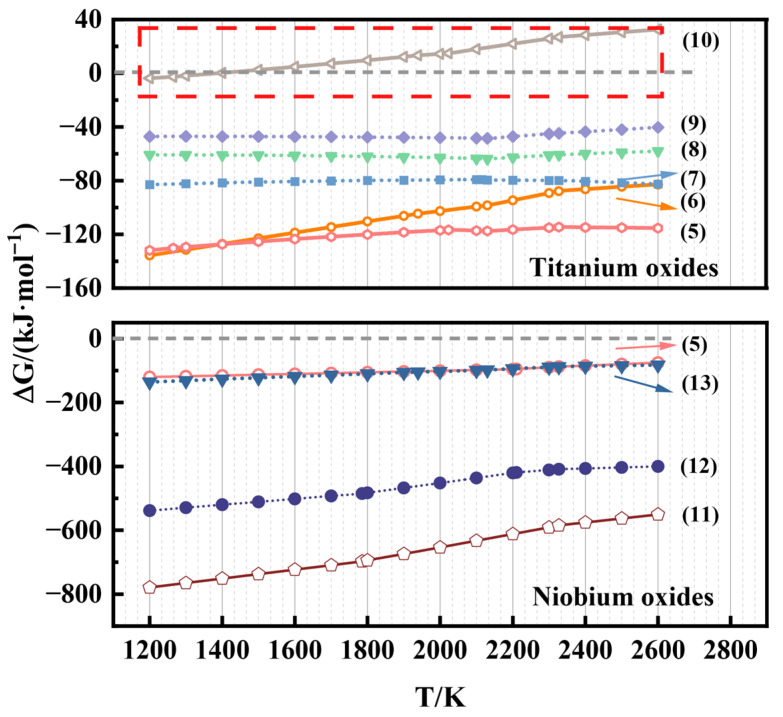
Plot of Gibbs free energy change with temperature for all reduction reactions.

As illustrated by the Gibbs free energy transformations of the main reduction reactions (Figure 3), except for Equation (10), the ΔG values for the reduction of all constituent oxides by Al are negative in the high-temperature region. From a thermodynamic perspective, this confirms the feasibility of their reduction. However, the magnitude of the driving force varies significantly among the different oxides. The ΔG values for the reduction of Nb-oxides are substantially more negative than those for TiO_2_ reduction. Notably, the ΔG for the NbO—Nb transformation is comparable to that of the initial TiO_2_—Ti step, suggesting that Nb_2_O_5_ possesses a much stronger thermodynamic affinity for aluminum.

Consequently, under conditions of competitive reduction with limited Al content, the reduction of niobium oxides is energetically favored to proceed more completely. In contrast, the ΔG values for the reduction of TiO_2_ to Ti and the intermediate TiO are closely aligned, implying that the further reduction of TiO to metallic Ti is the thermodynamically limiting step. Therefore, it can be inferred that in a restricted Al environment, the system tends to prioritize the full reduction of Nb-oxides, while a certain fraction of titanium may remain as lower-valence oxides (TiO) within the equilibrium products.

Theoretical calculations of the equilibrium phase compositions of the whole reaction system were carried out using the “Equilib” mod in Factsage 8.3, and the phase compositions and contents of each component were predicted at equilibrium when the total reaction reached equilibrium with different aluminum coefficients. The calculations were performed assuming an ideal adiabatic process (ΔH = 0).

To facilitate these calculations and the subsequent experiments, the specific mass ratios of the raw materials (TiO_2_, Al, Nb_2_O_5_, and CaO) designed under different ADCs are detailed in Table 2.

**Figure 4:**
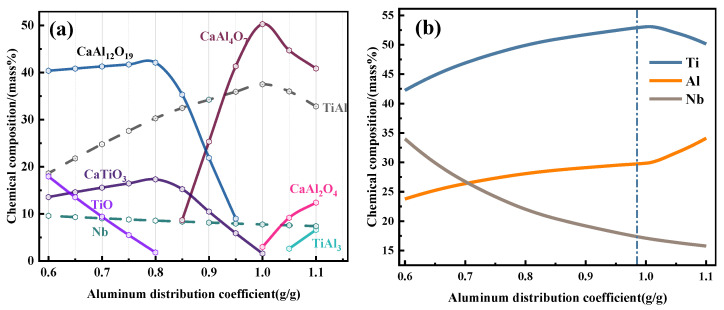
Variation trend diagrams of phase composition and alloy composition with ADC: (**a**) main phase composition; (**b**) chemical composition of alloy elements.

As shown in Figure 4a, in the thermodynamic system using Al as the reducing agent to reduce TiO_2_ and Nb_2_O_5_ and CaO as the slagging agent, the main alloy phases are TiAl, TiAl_3_, Nb, etc., and the main slag phases are CaAl_2_O_4_, CaAl_4_O_7_, and CaAl_12_O_19_, as well as unreduced TiO and CaTiO_3_ phases formed during the reaction. When the ADC is in the low range (0.6~0.8), the content of unreduced TiO in the system decreases with the increase in ADC, and the content of the corresponding TiAl phase increases. When the ADC is in the range of 0.8~1.0, Al reacts with CaTiO_3_ to form the CaAl_4_O_7_ phase with a higher melting point. When the ADC reaches 1.0, all TiO_2_ reacts with Al to form the TiAl phase. With the further increase in ADC, excess Al reacts with Ti to form the TiAl_3_ phase, and a low-melting-point CaAl_2_O_4_ phase precipitates at this time.

The equilibrium concentration of Ti, Al and Nb in the alloy was further analyzed, and the evolution trend is shown in Figure 4b. Nb_2_O_5_ is more easily reduced by Al than TiO_2_ in the system. Combined with the equilibrium phase concentration diagram, the reduction of Nb is more complete, and the transformation of TiO to Ti is the rate-limiting step of Ti reduction in the reaction system. Let ω_x_ (x = 0.6~1.1) be the reduction rate of Ti under different ADCs, then the content of unreacted residual Al in the system w_Al_ = W_Al_ × ω_x_. There is a ω_x_ correction factor between the actual non-equilibrium alloy composition and the equilibrium alloy composition, and the exact value of ω_x_ can be obtained by comparing with the subsequent experimental verification results.

**Figure 5:**
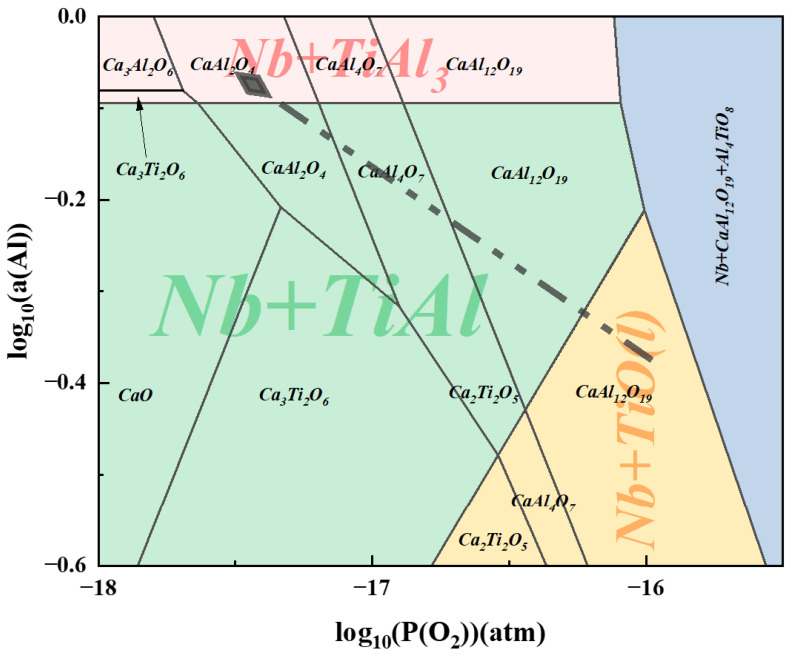
Integration of the dominant zones of the standard Ti-Al-Nb-O system as a function of O_2_ partial pressure and Al activity.

The dominant zone diagram of the system affected by O_2_ partial pressure and Al activity was plotted with the theoretical ADC of 1.0 as the standard, i.e., the molar ratio of Ca/(Ti + Ca + Nb) = 0.09 and Nb/(Ti + Ca + Nb) = 0.25 (Figure 5). The diagram intuitively shows the priority of Nb reduction: when the O_2_ partial pressure is between 1 × 10^−16^~1 × 10^−17^ atm, with the increase in Al activity in the system, Nb oxides are reduced first, followed by the gradual reduction of Ti oxides. With the further increase in Al activity, the alloy matrix in the system transforms from TiAl phase to TiAl_3_ phase accordingly, and the trend is shown by the dotted line in Figure 5.

### 3.2. Thermodynamic Evolution of Equilibrium Phases in Self-Propagating Reduction Processes

According to the actual design of the reaction system, considering that the reducing agent Al may be directly used as an alloying element to synthesize alloy products with a high Al content before it can fully reduce the metal oxides, the evolution of equilibrium phases at ADC of 0.6, 0.7, 0.8, 0.9 and 1.0 was investigated.

**Figure 6:**
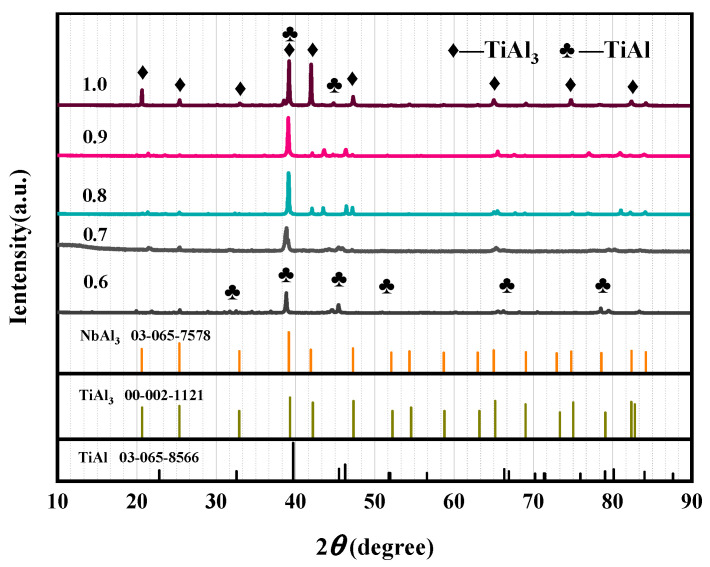
XRD patterns of alloys with different ADC.

As shown in Figure 6, the alloy is mainly composed of the TiAl phase and the TiAl_3_/NbAl_3_ phase. When the ADC is 0.6, the matrix phase of the alloy is TiAl, and the diffraction peak intensity of TiAl_3_ phase is very low, indicating insufficient Al content in the system. Combined with the theoretical prediction in Figure 2, it is inferred that when Al is used as the reducing agent, the reduction priority of Nb_2_O_5_ is much higher than that of TiO_2_ and low-valent Ti oxides. Therefore, the insufficient Al in the system preferentially reacts with Nb_2_O_5_, resulting in a low reduction rate of TiO_2_, low Al content in the metal phase, and an atomic ratio of Ti to Al close to 1:1.

With the increase in ADC, the proportion of Al available for the reduction of TiO_2_ and low-valent Ti oxides increases, which improves the reduction rate of Ti and increases the residual Al content in the equilibrium system. As shown in Figure 5, when the O_2_ partial pressure of the system is constant, the Al activity increases to saturation, and the Al_3_Ti phase begins to precipitate in the equilibrium system. The further reduction of Ti oxides also promotes the left shift in the oxygen partial pressure equilibrium, which accelerates the formation of the Al_3_Ti phase in the alloy.

When the ADC increases to 0.8 and 0.9, the diffraction peak intensity of Al_3_Ti in the alloy increases significantly. The strengthening mechanism of Nb on the Al_3_Ti phase shows that Nb atoms will occupy the Ti sublattice position in TiAl [33]. The diffraction patterns of Al_3_Ti and Al_3_Nb phases are basically coincidence, so it is speculated that the alloy is in a state where TiAl is the matrix phase and Al_3_Ti and Al_3_Nb coexist. When the ADC reaches 1.0, the reduction rate of Ti oxides is the highest, and Al is oversaturated in the high-temperature melt (corresponding to the Al_3_Ti region in Figure 5). Therefore, the matrix phase of the alloy is Al_3_Ti at this time, and the Al_3_Nb phase exists due to the replacement of Ti atoms by Nb. Free Ti at high temperature reacts with saturated Al to form part of the TiAl phase to reach a stable state, which is consistent with the thermodynamic prediction trend in the previous section.

**Figure 7:**
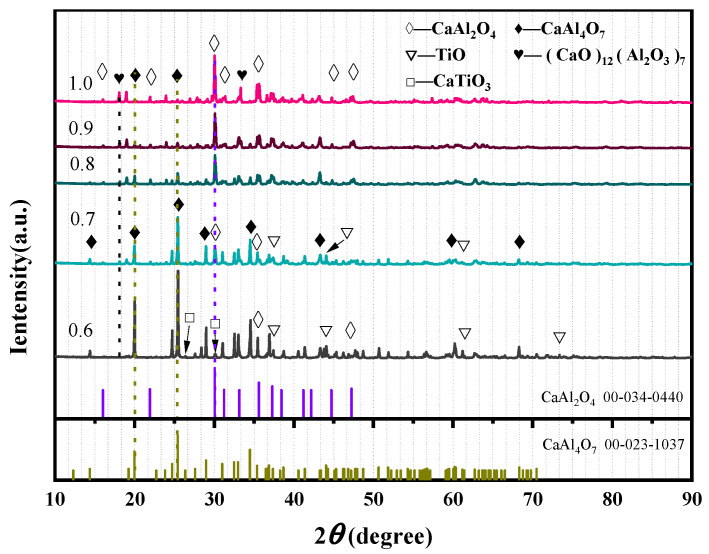
XRD patterns of slag with different ADCs.

As can be seen from Figure 7, the main constituent phases of the slag during the reduction process are CaAl_4_O_7_ and CaAl_2_O_4_. With the gradual increase in ADC, the diffraction peak intensity of CaAl_4_O_7_ decreases, while the diffraction peak intensity of CaAl_2_O_4_ increases, showing a negative correlation between the two, which is consistent with the thermodynamic equilibrium calculation results. When the ADC is low (0.6, 0.7), the unreduced TiO phase still exists in the slag. At this time, the Al_2_O_3_ content in the equilibrium slag system is low, and part of the titanium oxides form the CaTiO_3_ phase with CaO. With the gradual increase in Al content, TiO is more fully reduced, and the diffraction peak of the TiO phase in the slag is gradually weakened when the ADC is above 0.8. This is consistent with the law obtained by thermodynamic equilibrium calculation, that is, the rate-limiting step for the reduction of TiO_2_ to Ti is the process of TiO → Ti, and high ADC can promote the reduction of low-valent titanium oxides.

At the same time, the diffraction peak intensity of the CaTiO_3_ phase decreases gradually. With the further increase in ADC (0.9, 1.0), the main phase of the slag changes to the low-melting-point CaAl_2_O_4_ phase, accompanied by the appearance of the (CaO)_12_(Al_2_O_3_)_7_ phase with a lower melting point.

Compared with the thermodynamic phase equilibrium diagram, the relationship curve between the actual phase composition of alloy and slag and ADC shifts to the direction of high ADC. This is because part of TiO is not reduced in the actual reaction, and Al, which is designed as a reducing agent, rapidly synthesizes intermetallic compounds with Ti in the high-temperature molten state, resulting in the change in Al content in the alloy and the corresponding shift in the phase evolution trend. The TiO and CaTiO_3_ phases higher than the theoretical predicted value reduce the proportion of CaO in the slag system, leading to the overall right shift in the equilibrium concentration trend graph. It can be seen that the thermodynamic and kinetic competition of each reaction changes with the evolution of the reaction equilibrium phase during the actual reaction process.

### 3.3. Quality Characterization of Alloys

The target mass ratio of the Ti_45_Al_8_Nb (at%) alloy is Ti:Al:Nb = 54 wt.%:29 wt.%:17 wt.%. Al in the raw materials acts as both the reducing agent and the alloying element, and the two roles compete with each other during the reduction process. Therefore, the ADC has a critical impact on the composition control of the alloy.

**Figure 8:**
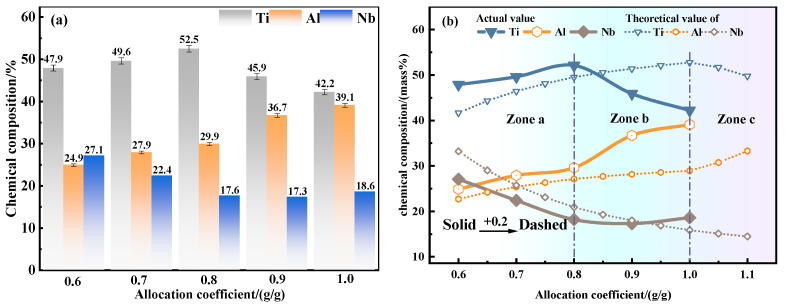
Chemical composition of the metallic elements in the synthesized alloys as a function of the ADC: (**a**) experimental elemental concentrations; (**b**) comparison and deviation analysis between the experimental results and theoretical calculations.

As shown in Figure 8, the chemical composition of the alloy is consistent with the thermodynamic theoretical prediction. With the increase in ADC, the Ti content first increases and then decreases, the Al content shows an upward trend, and the Nb content shows a downward trend. Due to the effect of the Ti reduction rate ω_x_ (x = 0.6~1.1) mentioned above, the actual evolution curve of alloying elements is shifted to the left by about 0.2 ADC points compared with the theoretical trend. The ADC range is divided into three zones: zone a (0.6~0.8), zone b (0.8~1.0), and zone c (1.0~1.1).

When the ADC is in zone a, the reducing agent Al is insufficient, and Al preferentially reacts with Nb_2_O_5_. Combined with the XRD patterns of the alloy and slag, the reduction of Ti is not complete in this zone, and part of Ti enters the slag phase in the form of TiO and CaTiO_3_. With the increase in Al content, the reduction degree of Ti increases, and more Ti and Al form intermetallic compounds and enter the alloy phase, so the contents of Ti and Al in the alloy increase gradually. When the ADC is 0.8, the Ti reduction rate of the system reaches the maximum value, and the Al content in the equilibrium system is close to saturation. Continuing to increase the Al content has little effect on the further reduction of TiO but plays an increasing role in the transformation of the alloy matrix phase from TiAl to Al_3_Ti.

That is, in zone b, most of the continuously increased Al enters the alloy phase in the form of alloying, the Al content in the alloy increases, and the Ti reduction rate reaches the maximum. Nb will replace Ti in Al_3_Ti to form the Al_3_Nb phase in zone b, which will inevitably lead to the formation of some Ti-enriched regions in the alloy. Zone c is the part of the thermodynamic theoretical process after Al supersaturation, which corresponds to zone b of the actual process. Combining the two offsets, the actual composition M_x_ ≈ the theoretical composition M_(x−0.2)_ (x is the ADC, x = 0.6~1.2). There is a fixed 20% lateral offset between the theoretical prediction curve and the actual composition curve, and the theoretical prediction curve will overlap with the actual composition curve after shifting to the left by 0.2. This offset is the kinetic effect in the system, that is, the Ti reduction rate ω_x_ = 0.8.

**Figure 9:**
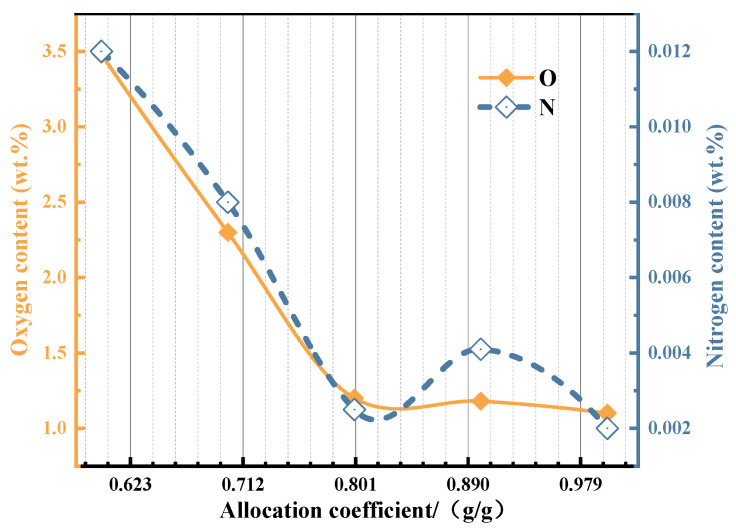
Contents of O and N in alloys with different aluminum alloys.

As illustrated in Figure 9, the ADC affects the oxygen (O) and nitrogen (N) contents in the synthesized alloy by influencing both the reduction rate of the metal oxides and the composition of the slag. The oxygen content (represented on the left axis) fluctuates between 1.0 and 3.5 wt.%, while the nitrogen content (represented on the right axis) ranges from 0.002 to 0.012 wt.%. Overall, both the O and N contents exhibit a noticeable downward trend as the ADC increases.

At lower ADCs (0.6 and 0.7), the amount of Al in the reaction system is insufficient. This leads to a higher retention of unreduced Ti oxides and a lower generation of Al_2_O_3_. Under these conditions, the predominant slag phase formed is the composite CaAl_4_O_7_, which possesses a relatively high melting point, accompanied by the formation of some CaTiO_3_. This high-melting-point slag hinders effective slag–metal separation, resulting in a loose slag structure and an increased quantity of residual non-metallic inclusions trapped within the alloy. Consequently, the overall oxygen and nitrogen impurity levels in the final alloy are elevated.

When the ADC increases to above 0.8, the oxygen content in the alloy is between 1.0 and 1.25 wt.%. At this time, the Ti reduction rate in the system is close to the limit, the main slag phase gradually transforms into low-melting-point slag dominated by CaAl_2_O_4_, the slag–metal separation effect is improved, and the content of inclusions in the alloy is reduced, so the oxygen content is maintained at a relatively low level.

**Figure 10:**
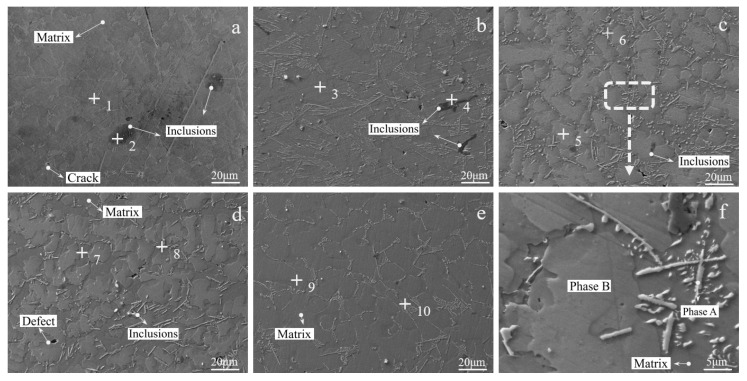
SEM images of alloys with different aluminum ratio ((**a**)—0.6; (**b**)—0.7; (**c**)—0.8; (**d**)—0.9; (**e**)—1.0; (**f**)—the box selection area in (**c**)).

It can be seen from Figure 10 and Table 3 that at a low ADC (a: 0.6), the alloy is dominated by the TiAl matrix phase, accompanied by large-size inclusions (~20 μm) with the main composition of Al_2_O_3_. The matrix phase has a high Ti content, and dendrite-like crystals precipitate on the matrix surface, which are Ti-enriched regions (point 1 in Figure 10a). The overall defect density of the alloy is high, and the slag–metal separation is incomplete.

As shown in Table 3, trace amounts of silicon (Si) were detected at some representative points of the alloy. This is primarily due to the use of industrial-grade raw materials, specifically the aluminum (Al) powder used as the reducing agent, which contains trace Si as an inherent impurity. During the intense self-propagating reduction–displacement reaction, these trace elements from Al are transitioned into the alloy matrix, leading to the minor Si content observed in the final product.

**Table 3:** Table of representative point-spot scanning energy spectrum analysis in SEM (wt%).

Serial Number		Ti	Al	Nb	O	Si
a	1	49.8	26.2	8.1	6.4	9.5
	2	38.1	42.7	13.0	6.2	0
b	3	0.4	52.2	0	47.4	0
	4	41.1	37.5	21.4	0	0
c	5	16.4	50.7	32.9	0	0
	6	52.4	27.1	20.6	0	0
d	7	42.3	51.8	4.8	0	0
	8	56.4	19.2	12.6	8.8	3.0
e	9	41.9	39.7	18.4	0	0
	10	70.4	18.0	11.5	0	0

With the increase in ADC (a: 0.6 → b: 0.7 → c: 0.8), the Al content in the alloy increases gradually, and irregular Al-enriched zones appear (point 5 in Figure 10c), which are a large number of Al_3_Ti/Al_3_Nb mixed phases combined with XRD results. At this time, the Ti/Al ratio of the alloy matrix phase decreases gradually, the transformation from TiAl phase to TiAl_3_ phase occurs, and the replacement of Ti atoms by Nb increases simultaneously. The replaced Ti precipitates and enriches in the dendritic grains on the matrix surface (Phase A in Figure 10f). The inclusions and defects in the alloy are significantly reduced, and the size is reduced to 5~8 μm.

**Figure 11:**
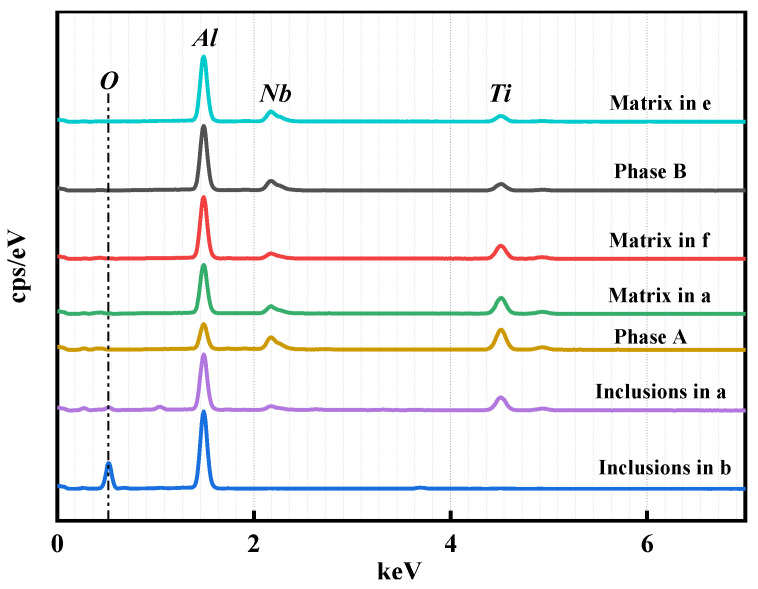
EDS spectra corresponding to Phase A and Phase B in the enlarged SEM image (Figure 10f).

When the ADC increases to 1.0 (e), the Al content of the alloy reaches 40 wt.%, and the matrix phase transforms into a composite phase of TiAl_3_ and NbAl_3_. Combined with EDS analysis, the surface dendritic grains are the replaced Ti-enriched regions, Nb forms the NbAl_3_ phase with Al, and the proportion and size of inclusions are further reduced with a homogeneous grain size. The enlarged SEM image of the alloy with an ADC of 0.8 (Figure 10f) combined with EDS results (Figure 11) shows that Phase B is the mixed zone of Al_3_Ti and Al_3_Nb, Phase A is the Ti-enriched zone, and the rest of the matrix phase is the TiAl phase.

**Figure 12:**
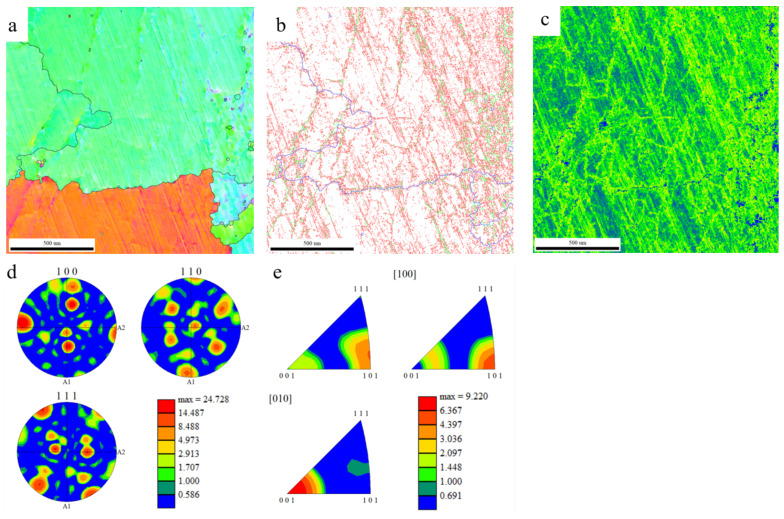
EBSD analysis of the alloy with ADC 0.8 ((**a**)—orientation imaging map; (**b**)—granular boundary distribution map; (**c**)—phase distribution map; (**d**)—polar map; (**e**)—antipolar map).

Figure 12 shows the EBSD analysis results of the alloy with an ADC of 0.8. The alloy prepared by in situ reduction has obvious grain coarsening characteristics, with grain size mainly distributed in the range of 800–1300 µm, and grains with a diameter larger than 1200 µm are dominant (Figure 12a). Low-angle grain boundaries (2–5°) are dominant, accounting for 83.1% of the total grain boundary length, while high-angle grain boundaries (>15°) only account for 3.9%, indicating that there are a large number of subgrain boundaries or deformation zones inside the material.

The inverse pole figure shows a wide distribution of crystal orientations (Figure 12e), and the pole figure and Euler angle cross-section reveal obvious texture characteristics (Figure 12d). In particular, the orientation in the <111> direction is concentrated in the [001] pole figure, with the maximum texture intensity as high as 102, indicating that the material formed a strong selective orientation during the reaction and solidification process. The average misorientation map shows an obvious orientation gradient inside the grains, which is consistent with the high proportion of low-angle grain boundaries, further confirming that the material underwent plastic deformation or recrystallization during the solidification process.

**Figure 13:**
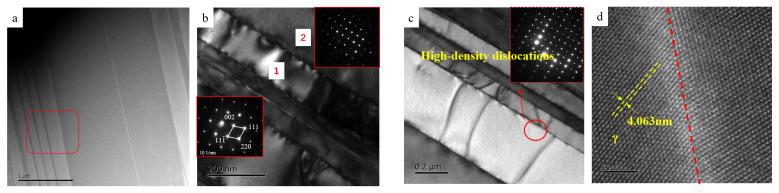
TEM images of 0.8 mated aluminum factor alloy ((**a**)—low magnification BF TEM image; (**b**)—high magnification BF TEM and 1, 2 region diffraction spot image; (**c**)—high magnification and diffraction spot image of phase interface; (**d**)—high resolution STEM image).

Figure 13 presents the transmission electron microscopy (TEM) characterization of the Ti-Al-Nb alloy, which exhibits a characteristic alternating lamellar microstructure. The low-magnification bright-field (BF) TEM image (Figure 13a) reveals a relatively uniform contrast background superimposed with periodic dark fringes, suggesting the presence of inherent structural or compositional variations within the lamellae. Further detailed examination using high-magnification BF TEM (Figure 13b) and the corresponding selected area electron diffraction (SAED) analysis confirms the coexistence of the tetragonal L1_0_-TiAl phase and the hexagonal D0_19_-Ti_3_Al phase [34]. The SAED patterns of these two constituent phases exhibit highly coincident diffraction spots, thereby indicating a strictly defined crystallographic orientation relationship between the Ti-enriched and Nb-enriched domains.

Further microstructural analysis (Figure 13c) reveals the presence of deformation twins along the lamellar interfaces of the banded structure. Notably, localized regions exhibit a high density of dislocations and stacking faults. While these defects may be partially attributed to mechanical stress during the sample preparation process, they primarily serve as a critical mechanism for accommodating internal thermal stresses and lattice mismatch between the distinct phases during cooling [35]. High-resolution TEM (HRTEM) observation of the phase boundary (Figure 13d) demonstrates that the lattice fringes traverse the interface continuously without significant misfit dislocations. This indicates the formation of a low-energy coherent or semi-coherent interface between the γ-TiAl phase and the L1_2_-Al_3_Nb phase. Overall, the three phases (TiAl, TiAl_3_, and Al_3_Nb) are distributed in an alternating lamellar or domain-like morphology. The excellent lattice matching and the resulting low interfacial energy are highly conducive to the microstructural stability of the alloy, which is expected to play a pivotal role in governing its high-temperature mechanical performance [36].

## 4. Conclusions

In this paper, a Ti_45_Al_8_Nb alloy with the target composition was synthesized in situ via a metallurgical thermodynamic design combined with aluminothermic self-propagating reduction, and the influence of ADC on the self-propagating reaction process was systematically investigated. The main conclusions are as follows:

(1) Thermodynamic design results show that the reduction priority of Nb_2_O_5_ to Nb is much higher than that of TiO_2_ to Ti, and the rate-limiting step for the complete reduction of TiO_2_ to Ti is the further reduction of TiO. With the increase in ADC, the alloy phase transforms from TiAl to TiAl_3_, and the slag system transforms from high-melting-point CaAl_4_O_7_ to low-melting-point CaAl_2_O_4_.
(2) The experimental results under different ADCs are consistent with the thermodynamic design trend. Due to the influence of kinetic factors on the reaction equilibrium in the actual reaction process, the equilibrium shifts positively. There is a fixed 20% lateral offset between the theoretically calculated value and the actual test value, that is, the Ti reduction rate ω_x_ = 0.8. The correction coefficient can be used to accurately predict the specific composition of the alloy through the raw material ratio.
(3) When the ADC of the alloy is 0.8, the mass fractions of Ti, Al, Nb, O and N are 51.8 wt.%, 29.5 wt.%, 17.4 wt.%, 1.2 wt.% and 0.0016 wt.%, respectively, which are close to the target composition. When the ADC is ≥0.8, the alloy has a homogeneous microstructure, low inclusion content with size less than 8 µm, and the Al content in the alloy is positively correlated with the coefficient. When the ADC is too low, the alloy yield is low and the inclusion separation effect is poor.
(4) The main inclusion in the alloy is Al_2_O_3_, and the alloy phases are the TiAl matrix phase, the blocky Al_3_Nb phase and dendritic Ti-enriched zones. At the ADC of 0.8, the titanium–aluminum–niobium alloy forms a coarse-grained structure dominated by grains larger than 1200 µm, with a strong texture dominated by the <111> orientation, containing 83.1% of low-angle grain boundaries, and the texture intensity is higher than 102. The alloy has an obvious orientation gradient, indicating that significant plastic deformation and recrystallization occurred during the solidification process. The titanium–aluminum–niobium alloy shows an alternating banded microstructure, and the three phases of TiAl, TiAl_3_ and Al_3_Nb form a low-energy coherent interface between the Ti-enriched and Nb-enriched regions, which jointly enhances the structural stability of the alloy.

## Figures and Tables

**Table 1:** Specifications and manufacturers of the raw materials used in this study.

Ingredient	Granularity	Purity	Manufacturer
TiO_2_	0.1~0.3 μm	99.5	Jinzhou Pengda Titanium Dioxide Manufacturing Co., Ltd., Jinzhou, China
Nb_2_O_5_	0.1~0.2 μm	99.9	Tianjin Zhiyuan Chemical Reagents Co., Ltd., Tianjin, China
Al	≤3 mm	99.5	CITIC Jinzhou Metal Co., Ltd., Jinzhou, China
KClO_3_	≤2 mm	99.5	Xilong Science Co., Ltd., Guangzhou, China
CaO	≤2.5 mm	99.5	Tianjin Zhiyuan Chemical Reagents Co., Ltd., Tianjin, China

**Table 2:** Material ratios with different ADCs.

ADC	m(TiO_2_):m(Al): m(Nb_2_O_5_):m(CaO)	ADC	m(TiO_2_):m(Al): m(Nb_2_O_5_):m(CaO)
1.1	1.05:1:0.28:0.36	0.8	1.45:1:0.39:0.36
1.05	1.10:1:0.30:0.36	0.75	1.54:1:0.42:0.36
1	1.16:1:0.31:0.36	0.7	1.65:1:0.45:0.36
0.95	1.22:1:0.33:0.36	0.65	1.78:1:0.48:0.36
0.9	1.29:1:0.35:0.36	0.6	1.93:1:0.52:0.36
0.85	1.36:1:0.37:0.36		

## Data Availability

The original contributions presented in this study are included in the article/Supplementary Material. Further inquiries can be directed to the corresponding authors.

## Supplementary Materials

The following supporting information can be downloaded at: https://www.mdpi.com/article/10.3390/ma19091689/s1.

## References

[1] Xie X. (2004). Application and Development of High Temperature Materials in China. Mech. Eng. Mater..

[2] Wang L., Wang Y., Li X., Wu Y.W., Ding N., Wang N.B., Xin X.H., Su B.L. (2025). Research Progress and Application Status of Plastic Processing Technology for Titanium and Titanium Alloys in China. Titan. Ind. Prog..

[3] Zhang B., Li H., Hu H., Wang Q., Liu Y., Chen J. (2025). Performance Analysis and Application Fields of New Titanium Alloy Materials. Forg. Stamp..

[4] Lian C., Yang S., Zhou H., Liu J., Chen F., Zhao Y.Q. (2013). Effects of Alloying Elements Nb, Ta, Fe and Zr on Microstructure and Properties of Titanium Alloys. Hot Work. Technol..

[5] Mohammed K.E., Olivier D., Gervais S. (2021). Titanium: An Overview of Resources and Production Methods. Minerals.

[6] Chen G.L., Peng L.M., Qi Z.J., Wang Y.L. (2001). High Nb containing TiAl base alloys. Intermetallics.

[7] Choi W., Jourdan J., Matveichev A., Jardy A., Bellot J.-P. (2017). Kinetics of Evaporation of Alloying Elements under Vacuum: Application to Ti Alloys in Electron Beam Melting. High Temp. Mater. Process..

[8] Clemens H., Kestler H. (2000). Processing and applications of intermetallic γ-TiAl-based alloys. Adv. Eng. Mater..

[9] Li S., Zhang H., Jiang H., Yao Z.H., Dong J.X. (2026). Process Optimization and Control of Vacuum Consumable Arc Melting for Super Large GH4169 Ingots. Acta Aeronaut. Astronaut. Sin..

[10] Wang G., Yang J., Li X. (2017). Microstructure and mechanical properties of a Ti–22Al–25Nb alloy fabricated from elemental powders by mechanical alloying and spark plasma sintering. J. Alloys Compd..

[11] Joan F.L., Raúl E.P., Vicente B.A. (2023). Powder Metallurgy: A New Path for Advanced Titanium Alloys in the EU Medical Device Supply Chain. Metals.

[12] Bououdina M., Guo Z.X. (2002). Characterisation of structural stability of (Ti(H_2_)-22Al-23Nb) powder mixtures during mechanical alloying. Mater. Sci. Eng. A.

[13] Chen G.L., Xu X.J., Teng Z.K., Wang Y.L., Lin J.P. (2007). Microsegregation in high Nb containing TiAl alloy ingots beyond laboratory scale. Intermetallics.

[14] Li S.J., Wang Y.L., Lin J.P., Che G. (2004). Preparation technology of high niobium titanium aluminum alloy. J. Aeronaut. Mater..

[15] Li D.H., Wang B.B., Luo L.S., Li X., Yu J., Wang L., Xu Y., Su Y., Guo J., Fu H. (2022). In-situ synthesis of Al_2_O_3_-reinforced high Nb–TiAl laminated composite with an enhanced strength-toughness performance. Ceram. Int..

[16] Wang X., Tang Y., Quan M.H., Zheng L., Meng Y. (2022). Formation of columnar strengthening phases and mechanical properties of Nb–Ti–Al alloy by spark plasma sintering. Mater. Sci. Eng. A.

[17] Jabbar H., Monchoux J.-P., Houdellier F., Dollé M., Schimansky F.-P., Pyczak F., Thomas M., Couret A. (2010). Microstructure and mechanical properties of high niobium containing TiAl alloys elaborated by spark plasma sintering. Intermetallics.

[18] Yang F., Kong F.T., Chen Y.Y., Xiao S.L. (2010). Effect of spark plasma sintering temperature on the microstructure and mechanical properties of a Ti_2_AlC/TiAl composite. J. Alloys Compd..

[19] Chen Y.Y., Yu H.B., Zhang D.L., Chai L.H. (2009). Effect of spark plasma sintering temperature on microstructure and mechanical properties of an ultrafine grained TiAl intermetallic alloy. Mater. Sci. Eng. A.

[20] Wang J., Wang Y., Liu Y., Li J., He L., Zhang C. (2015). Densification and microstructural evolution of a high niobium containing TiAl alloy consolidated by spark plasma sintering. Intermetallics.

[21] Xun Y., Mohamed F.A., Lavernia E.J. (2004). Synthesis of nanocrystalline Zn–22 Pct Al using cryomilling. Metall. Mater. Trans. A.

[22] Lahiri A., Ghosh A., Roy S., Borbély G., Inoue T., Takeyasu K. (2009). Electrodeposition of Al_3_Ti intermetallic from ionic liquid at room temperature. Electrochem. Commun..

[23] Chen G.Z., Fray D.J., Farthing T.W. (2000). Direct electrochemical reduction of titanium dioxide to titanium in molten calcium chloride. Nature.

[24] Sun F., Rojas P., Zúñiga A., Lavernia E.J. (2006). Nanostructure in a Ti alloy processed using a cryomilling technique. Mater. Sci. Eng. A.

[25] Levashov E.A., Mukasyan A.S., Rogachev A.S., Shtansky D.V. (2017). Self-propagating high-temperature synthesis of advanced materials and coatings. Int. Mater. Rev..

[26] Song Y.L., Dou Z.H., Zhang T.A. (2020). Preparation of Ti-Al alloy by metal thermal reduction method. Rare Met. Mater. Eng..

[27] Liang Y.J., Che Y.C. (1993). Handbook of Inorganic Thermodynamic Data.

[28] Munir Z.A., Holt J.B. (1989). Combustion and Plasma Synthesis of High Temperature Materials.

[29] Mossino P. (2004). Some aspects in self-propagating high-temperature synthesis. Ceram. Int..

[30] Merzhanov A.G. (1990). Combustion and Plasma Synthesis of High-Temperature Materials.

[31] Borovinskaya I.P., Merzhanov A.G., Novikov N.P., Filonenko A.K. (1974). Gasless combustion of mixtures of powdered transition metals with boron. Combust. Explos. Shock. Waves.

[32] Merzhanov A.G. (1994). Solid flames: Discoveries, concepts, and horizons of cognition. Combust. Sci. Technol..

[33] Voisin T., Monchoux J., Durand L., Karnatak N., Thomas M., Couret A. (2015). An innovative way to produce γ-TiAl blades: Spark plasma sintering. Adv. Eng. Mater..

[34] Tao H.J., Zhou S., Liu Y., Yin J., Xu H. (2017). First-principles study on the concentration and interaction of point defects in D0_19_-Ti_3_Al. Acta Metall. Sin..

[35] Merzhanov A.G. (1995). Fluid dynamics phenomena in the processes of self-propagating high-temperature synthesis. Combust. Sci. Technol..

[36] Appel F., Paul J.D.H., Oehring M. (2011). Gamma Titanium Aluminide Alloys: Science and Technology.

