# Supplementary material for: Metallurgical Thermodynamic Design Research on the In Situ Synthesis of Ti-Al-Nb Alloys Using Thermit Self-Propagating Reduction
Source: Materials (Basel). 2026 Apr 22;19(9):1689. doi: 10.3390/ma19091689 (PMC13163873; doi:10.3390/ma19091689)
Supplement: Supplementary file 1 [file materials-19-01689-s001.zip › materials-4254802-supplementary.pdf]

In the thermodynamic calculation of the adiabatic temperature of self-propagation, the main calculation focuses on the heat release of the self-propagating system. Throughout the entire thermodynamic calculation process, the following assumptions are made for the system:

(1) The system is in an ideal adiabatic state with no energy exchange with the external environment;

(2) The system reaction proceeds in accordance with the main reaction without any side reactions;

(3) The reaction is complete with no residual reactants.

Based on the above three assumptions, the adiabatic temperature can be calculated via thermodynamic equations using the heat capacity and enthalpy related to substance conversion and phase transition (when phase transition occurs) in the chemical equation of the self-propagating reaction.

For the self-propagating reaction system: A(s)+B(s)=AB(s)+ΔH, the heat released during this reaction process can be calculated by the following formula:

$$\Delta H = \Delta H_{298}^{\theta} + \int_{298}^{T_{ad}} \Delta c_p \mathrm{dT} \quad \text{(S1)}$$

In the formula: - System enthalpy. Assuming the system is in an ideal adiabatic state, this value is set to 0 kJ·mol⁻¹;

ΔH_{298}^{θ} Standard molar enthalpy of reaction at 298 K, kJ·mol⁻¹; Difference in constant-pressure heat capacity of the system, J·K⁻¹·mol⁻¹.

A1, A2, A3 and A4 are obtained by referring to the thermodynamic manual, and the following formula is used for approximate calculate c_p:

$$c_p = A_1 + A_2 \times 10^{-3} T + A_3 \times 10^{5} T^2 + A_4 \times 10^{-6} T^2 \quad \text{(S2)}$$

The adiabatic temperature is calculated under the following three conditions:

(1) When the adiabatic temperature is lower than the melting point Tm of the product:

$$-\Delta H_{298}^{\theta} = \int_{298}^{T_{ad}} \Delta c_p \mathrm{dT} \quad \text{(S3)}$$

(2) When the adiabatic temperature equals the melting point of the product (Tad=Tm):

$$-\Delta H_{298}^{\theta} = \int_{298}^{T_{ad}} \Delta c_p \mathrm{dT} + \gamma \Delta H_m \quad \text{(S4)}$$

Where: γ - Percentage of molten product, %; ΔHm - Melting heat of the product, kJ·mol⁻¹.

(3) When the adiabatic temperature is higher than the melting point Tm of the product:

$$-\Delta H_{298}^{\theta} = \int_{298}^{T_{ad}} \Delta c_p dT + \Delta H_m + \int_{T_m}^{T_{ad}} \Delta C_p dT \quad \text{(S5)}$$

Materials	△Hθ /kJ×mol-1	△Gθ /kJ×mol-1	T/K	a	b	c	Tem scope / K
Al	0	10.71<br>290.78	933	31.376<br>31.748<br>20.799	-16.393	-3.607	298-933<br>933-2767<br>2767-3200

Al_2O_3	-1675.27	118.41	2327	103.851	26.267	-29.091	298-800
				120.516	9.192	-48.367	800-2327
				144.863			2327-3500
Ti	0	4.14	1933	22.158	10.284		298-1155
		18.62		19.828	7.924		1155-1933
				35.564			1933-3000
TiO_2	-944.75	66.94	2143	62.856	11.36	-9.958	298-2143
				87.864			2143-3000
CaO	-634.29	79.50	2888	49.622	4.519	-6.945	298-2888
				62.76			2888-3500
$CaO\cdot 2Al_2O_3$	-3994.05			258.236	40.083	-64.015	298-2023
KCl	-436.68	26.28	1044	40.016	25.468	3.648	298-1044
				73.597			1044-1710
Nb	0	-10.85	2740	23.723	4.017		298-2740
				33.472			2740-5007
Nb_2O_5	-190205	-1942.90	1785	154.390	21.422	-25.522	298-1785
				242.254			

The overall equation for the reactions involved in the system is
$TiO_2+0.085Nb_2O_5+2.574Al=Ti+0.957Al+0.17Nb+0.809Al_2O_3$ (S6)

The adiabatic temperature (T_{ad}) for the self-propagating system is derived based on the principle of energy conservation. Under ideal adiabatic conditions, the standard reaction heat released at 298 K(ΔH^{θ}_{298}) is entirely consumed to heat the products to T_{ad}, including the latent heat of any phase transitions. For the target reaction:

First, the standard molar enthalpy of reaction at 298 K is calculated from the standard enthalpies of formation (ΔH^{θ}_{f}) of the constituents:

$$\Delta H^{\theta}_{298}=\Sigma\left(n\cdot\Delta H^{\theta}_{f}\right)_{products}-\Sigma\left(n\cdot\Delta H^{\theta}_{f}\right)_{reactants} \quad (S7)$$

Substituting the thermodynamic data

($\Delta H^{\theta}_{f}(TiO_2)$=-944.75 kJ/mol, $\Delta H^{\theta}_{f}(Nb_2O_5)$=-1902.05 kJ/mol, $\Delta H^{\theta}_{f}(Al_2O_3)$=-1675.27 kJ/mol), the total heat released by the system is determined to be $Q_{release}=-\Delta H^{\theta}_{298}=248.869$ kJ.

The total heat absorbed by the products is the sum of their individual sensible and latent heats:

$$\begin{gathered}Q_{Ti}=\int_{298}^{1155} C_{p,Ti(\alpha)}dT+\Delta H_{tr}+\int_{1155}^{1933} C_{p,Ti(\beta)}dT+\Delta H_{m(Ti)}+\int_{1933}^{T_{ad}} C_{p,Ti(l)}dT \\ Q_{Al}=0.957\times\left[\int_{298}^{933} C_{p,Al(s)}dT+\Delta H_{m(Al)}+\int_{933}^{T_{ad}} C_{p,Al(l)}dT\right] \\ Q_{Nb}=0.17\times\int_{298}^{T_{ad}} C_{p,Nb(s)}dT \\ Q_{Al_2O_3}=0.809\times\left[\int_{298}^{800} C_{p,Al_2O_3(s1)}dT+\int_{800}^{T_{ad}} C_{p,Al_2O_3(s2)}dT\right]\end{gathered} \tag{S8}$$

Finally, applying the heat balance equation $Q_{release}=Q_{Ti}+Q_{Al}+Q_{Nb}+Q_{Al_2O_3}$ and substituting the piecewise specific heat capacity polynomials, the resulting integral equation is solved numerically. By calculation, the equation balances exactly when the upper limit temperature **is 2103 K .** Thus, the adiabatic temperature for this reaction is established as $\mathbf{T_{ad}=2103\ K}$.
